# Wild edible plants and mushrooms of the Bamenda Highlands in Cameroon: ethnobotanical assessment and potentials for enhancing food security

**DOI:** 10.1186/s13002-020-00362-8

**Published:** 2020-03-04

**Authors:** Evariste Fedoung Fongnzossie, Christine Fernande Biyegue Nyangono, Achille Bernard Biwole, Patricia Nee Besong Ebai, Nina Bisi Ndifongwa, Jannet Motove, Siegfried Didier Dibong

**Affiliations:** 1 Advanced Technical Teacher’s Training School (ENSET), University of Douala, PO BOX 1872, Douala, Cameroon; 2 Faculty of Science, Department of Plant biology, University of Douala, PO BOX 24157, Douala, Cameroon

**Keywords:** Ethnobotanical knowledge, Wild edible plants and mushrooms, Bamenda Highlands, Food security

## Abstract

**Background:**

In seasons of food shortage, local communities across Africa use wild edible plants and mushrooms (WEPM) that contribute significantly to food security by supplementing households’ diets and providing alternative income. In the Bamenda Highlands of Cameroon, their biodiversity is believed to be rapidly declining as a result of land use change. Despite their potential beneficial values, there has been only limited research on this topic in this area. This study aims to document traditional knowledge related to the use of plants and mushrooms for food purpose by indigenous people of the Bamenda highland.

**Method:**

Ethnobotanical surveys were conducted in 6 localities (Mbengwi, Bafut, Nkwen, Mankon, Bambili, and Widikum) of the Bamenda Highlands of Cameroon, and 121 individuals were interviewed on commonly gathered and eaten WEPMs and their perception on their availability. Respondents were permanent residents selected based on their willingness to participate in the study. Specimens of recorded plants were collected and processed for future identification at the National Herbarium of Cameroon. Their nutritional potentials are discussed based on available literature.

**Results:**

A total of 47 species were recorded including leafy vegetable, spices, fruits, roots/tubers, and mushrooms. The top 5 most frequent are *Amaranthus* sp. (6.6%), *Termitomyces clypeatus* (6.4%), *Irvingia gabonensis* (5.2%), *Ricinodendron heudelotii* (5.1%), and *Aframomum* sp. (4.5%). Leafy vegetable and spices are the most diversified group with 13 species each. All recorded species are important from nutritional and pharmaceutical points. However, many of their values remain uninvestigated, while their natural populations are facing threats of degradation.

**Conclusion:**

WEPMs have great potential to contribute to food and nutritional security in the study area. Sound nutrients and metabolites profiling of poorly known species can enhance their contribution in addressing food insecurity.

## Introduction

Wild edible plants and mushrooms (WEPMs), according to the Food and Agriculture Organization (FAO), can be defined as “plants and mushrooms that grow spontaneously in self-maintaining populations in natural or seminatural ecosystems and can exist independently of direct human action” [1]. Historically, they have been important dietary components for most societies, and the species and mode of use have evolved in response to local contexts, preferences, and cultures. Since decades, scientists from all over the world have been investigating on wild edible plant resources which are identified to contribute significantly to food security by supplementing households’ diets in times of food scarcity and by providing some rare nutrients [1–5]. There are evidence from various studies across Africa indicating that wild fruits can supplement the daily diet and substitute for exotic fruits [6]. They are also seen as a particularly important way that households in rural Africa can improve their resilience to environmental change [7].

Cameroon is an ecologically diverse country endowed with high level of biodiversity. The country’s natural ecosystems are home to a wide variety of fauna, including 250 species of mammals, 542 fish, 848 birds, 330 reptiles, and 200 amphibians [8]. The country’s flora is estimated to comprise about 10,000 species, of which 7,850 have already been documented at the National Herbarium of Cameroon [9]. The importance of wild plants in feeding rural populations is very widely recognized through various studies. We can cite the work on a synthesis of knowledge on edible forest fruit trees in Cameroon [10], the study conducted on wild edible plants used by Guiziga people of far North Region of Cameroon [11], the study on indigenous edible fruits in sahelian domain of Cameroon [12], and several studies on non-timber forest products recording many plant species used for various purposes including as food [13–22]. Ethnobotanical surveys conducted among 102 sellers of edible wild plants based in 13 markets in the Yaounde City recorded 29 wild edible plant species used in 32 different food recipes [23]. A survey conducted in 4 popular markets of Douala reported a total of 25 wild edible fruit species sold in 4 popular markets of Douala (Littoral Region of Cameroon) [24].

The Bamenda Highlands contain the largest remaining patches of Afromontane forest in Central Africa, consisting of a mosaic of mixed gallery forest, Raphia forests, and savannah grasslands with some of the highest levels of endemism [25]. Previous ethnobotanical surveys showed that traditional societies in this area have always exploited wild edible plants that play a significant role in nutrition, food security, and income generation [26–29]. However, despite their abundance and varied potential beneficial values, wild edible plants and mushrooms have not received much attention similar to domesticated foods, yet they are increasingly seen by most researchers as an important alternative or complementary source of supply to deal with the needs in rural areas. A great majority of these studies have focused on medicinal species, and little emphasis has been paid to wild edible plants. In the Bamenda Highlands, during the last century, major social transformations and the rural exodus causing the concentration of population in large cities, has led to the disappearance of much of the knowledge and traditional practice of collecting spontaneous plants of food interest. Increased forest degradation driven by agriculture and pastoral development is threatening their biodiversity leading to growing poverty and increased vulnerability to food insecurity in many rural communities. However, today we are witnessing worldwide “rediscovery” of wild medicinal and food plants. This ethnobotanical knowledge of wild food plants exist mainly in rural communities that preserve traditional uses. This study proceeded through a substantial investigation of both the present practices and the oral history of the past few decades on traditional utilization of wild edible plants and mushrooms in the study area.

The present investigation was designed and carried out in the Bamenda Highlands of Cameroon to identify and categorize available wild edible plants and mushrooms, assess local perception on their availability, as well as their importance to improve food security among households in the area. We hypothesized that WEPM in this area are important source of nutrients with potentials to improve household’s food security.

## Materials and Methods

### Study site

The North-West Region of Cameroon forms the heart of the Bamenda Highlands, lying between latitude 5° 4′ and 7° 15′ north and longitude 9° 30′ and 11° 15′ east.

This area has a high human population density of approximately 100–250 people per square kilometer [30]. As a result, human pressure on natural ecosystems over the last century has been the cause of much biodiversity degradation.

The general climate of North-West Region has a rainy season between April and September and a dry season between October and March. Average rainfall is about 2400 mm and temperature average 23 °C, ranging between 15–32 °C [31]. This abundant rainfall contributes to the development of agriculture and forest regeneration. This area is known to support high levels of biological diversity and endemism. Three kinds of vegetation are present: lowland forest, mountain forest, and afro-alpine vegetation. The main ethnic groups in the area are of Tikar origin, and agriculture is their main occupation.

**Fig. 1:**
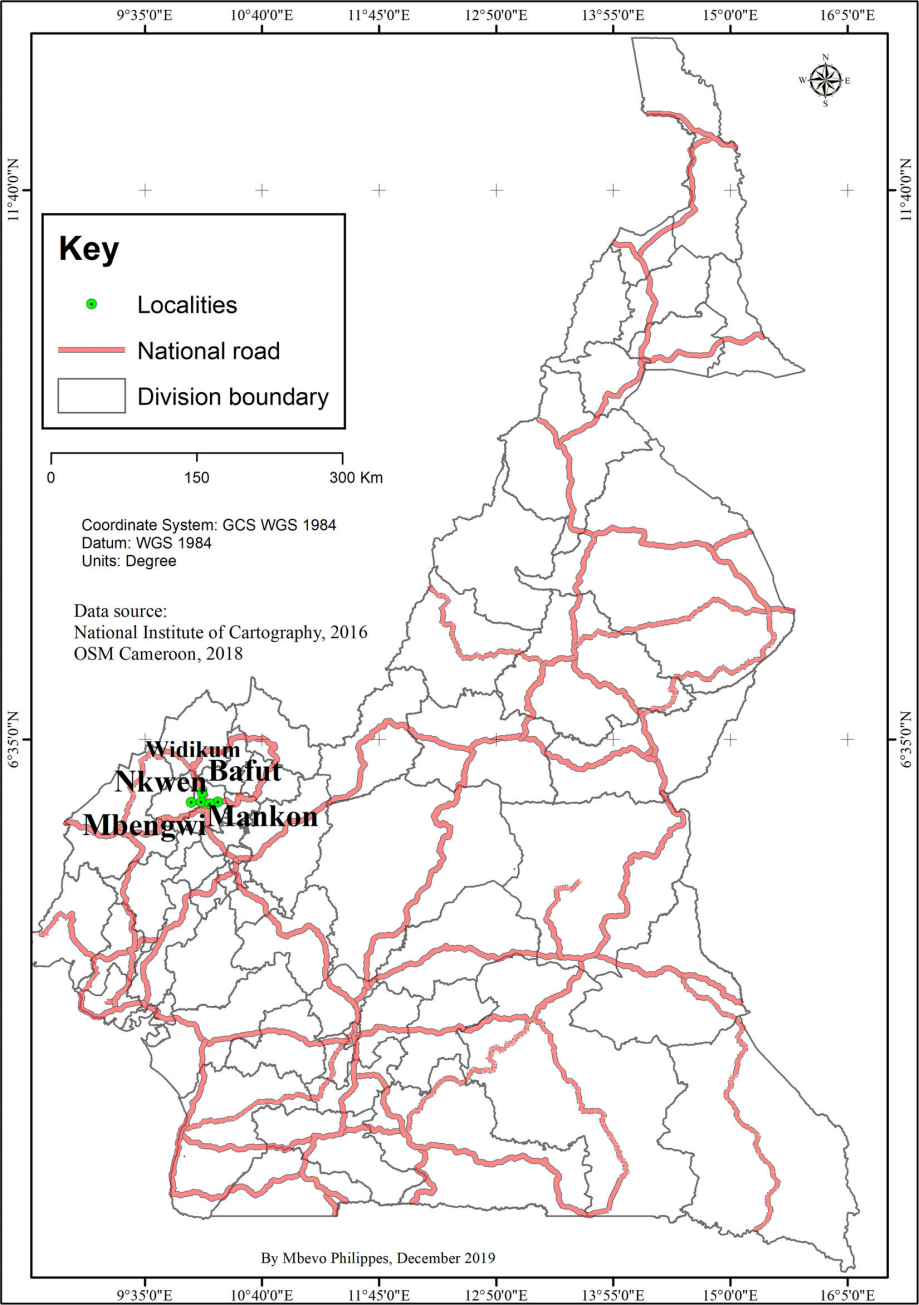
Location of the study area

The surveys were carried out in six villages selected based on the accessibility and availability of local informants. These are Mbengwi, Bafut, Nkwen, Mankon, Bambili, and Widikum (Fig. 1).

### Sampling procedure

Respondents were permanent residents and were selected based on their willingness to participate in the study. Our survey took place during the period March–April 2017. Overall, 121 individuals were interviewed (Table 1).

**Table 1:** Sociodemographic characteristics of respondents

Characteristics	Number	Percentage
Distribution by villages		
Mbengwi	30	24.8
Widikum	30	24.8
Bafut	16	13.2
Nkwen	15	12.4
Mankon	18	14.9
Bambili	12	9.9
Distribution by gender		
Female	45	37.2
Male	76	62.8
Distribution by age group		
Below 20	4	3.3
20–30	79	65.3
31–40	32	26.4
41–50	6	5

### Data collection and analysis

In this study, we followed standard methods in ethnobotanical researches to record the local knowledge on wild edible plants using interviews and field observations. A predesigned questionnaire was used to record the data. Before a formal interview was conducted, verbal or prior informed consent was sought and obtained from the concern village chief or chairman as well as the concerned individual informants by briefing clearly about the objectives of the study to them.

Overall, 121 individuals were interviewed on commonly gathered and eaten wild foods of plant origin (fruits, roots/tubers, mushrooms, leafy vegetables, spices, and others), their availability and conservation status. For each record, information gathered included local name of the plant, part used, and informant’s perception on availability of the species. Interviews were mostly conducted in the evenings when most people were back from their various occupations.

After the data collection, the data were organized based on the research questions. Based on their use, the recorded species were classified into different categories namely leafy vegetable, spices, mushroom, roots, tubers, and beverages. The taxonomic richness of each category was determined, and frequency of use citations of species was calculated.

### Plants specimen collection and identification

Voucher specimens of recorded WEPM were collected during field survey to different villages, allotted collection number, and pressed for future identification when returned from the field using various floras of Cameroon. These identifications were confirmed by the National Herbarium of Cameroon. Voucher specimens are kept at the herbarium of the Department of Plant Biology of the University of Douala.

## Results

### Consumption habits of wild edible plants

**Fig. 2:**
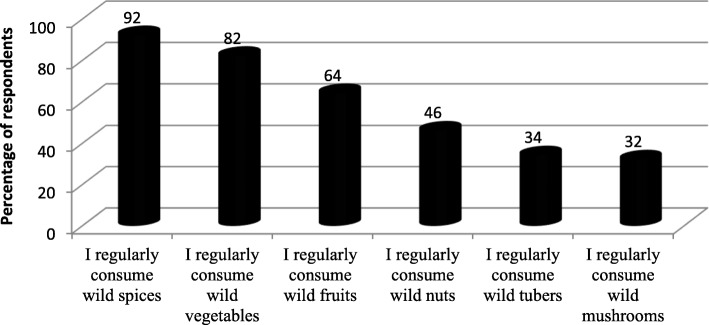
Citation frequency of consumption habits wild edible plants and mushrooms

Of the 121 people interviewed, it appeared that 77.5% of respondents agreed to consume wild edible plants and mushrooms. Among the eaten wild plant species, spices are the most consumed, and tubers are the least consumed (Fig. 2). From analysis, 85% of them agree that they are important contribution during difficult times of food shortage.

### Taxonomic diversity of wild edible plants and mushrooms

A total of 47 species were recorded (Table 2). They include leafy vegetable (13 species), spices (13 species), fruits (12 species), mushroom (6 species), roots and tubers (4 species), and beverages (1 species). The top 10 most frequently cited are *Amaranthus* sp. (6.6%), *Termitomyces clypeatus* R. Heim (6.4%), *Irvingia gabonensis* (Aubry-Lecomte ex O'Rorke) Baill. (5.2%), *Ricinodendron heudelotii* (H.E.Baillon) J.B.Pierre ex E.M.Heckel. (5.1%), *Aframomum* sp. (4.5%), *Raphia farinifera* (Gaertn.) Hyl. (4.2%), *Termitomyces letestui* (Pat.) Heim. (4.1%), *Termitomyces* sp. (4%), *Xylopia aethiopica* (Dunal) A. Rich. (3.8%), and *Afrostyrax lepidophyllus* Mildbr. (3.3%).

**Table 2:** Taxonomic list of the recorded species

Local name	Locality/language	Scientific name	Family	Category	Voucher specimen	Citation frequency
*Masangha*, *marsareh*	Mendakwe	*Acanthaceae*	Acanthaceae	Vegetable	FFE027	0.8
*Frechooh*	Widikum	*Aframomum sp*.	Zingiberaceae	Fruit	FFE011	4.5
*Alelock*, *Echap*	Be’feu-Bafut	*Afrostyrax lepidophyllus* Mildbr**.**	Huaceae	Spice	FFE016	3.3
*Contry green*, *Etang*	Pidgin, Mbengwi	*Amaranthus hybridus* L.	Amaranthaceae	Vegetable	FFE0026	2.1
*Contri green*, *Etang*	Pidgin, Mbengwi	*Amaranthus sp*.	Amaranthaceae	Vegetable	FFE018	6.6
*Ekarebang*, *ekereket*, *ngwetuat*	Bambili	*Annona muricata* L.	Annonaceae	Fruit	FFE012	3.2
*Black*, *Gejabe*	Mankon-Alakuma	*Canarium schweinfurthii* Engl.	Burseraceae	Fruit	FFE019	1.2
*Cola*, *tamtsi*	Wum	*Cola acuminata* (*P*. *Beauv*.) Schott and Endl	Sterculiaceae	Fruit	FFE037	1.3
*Cola*, *tamtsi*	Bafut	*Cola anomala* K. Schum.	Sterculiaceae	Fruit	FFE050	2.3
*Monkey Cola*, *awulawela*	Muganka	*Cola lepidota* K. Schum	Sterculiaceae	Fruit	FFE034	2.1
*Lemgambelle*	Bali Nyonga	*Corchorus olitorius* L.	Malvaceae	Vegetable	FFE014	0.5
*Monkey sugarcane*	Bafut	*Costus afer* Ker Gawl.	Costaceae	Roots/tubers	FFE025	0.4
*Ecute/bush yams*	Bafut	*Dioscorea* spp.	Dioscoreaceae	Roots/tubers	FFE021	0.3
*Bitter cola/okogon*	Bali Nyonga	*Garcinia kola* Heckel	Clusiaceae	Fruit	FFE039	2.2
*Eru*	Santa	*Gnetum* spp.	Gnetaceae	Vegetable	FFE042	1.6
*Mfume*, *njap*, *njap*, *bush mango*	Pidgin-Furawa	*Irvingia gabonensis* (Aubry-Lecomte ex O'Rorke) Baill.	Irvingiaceae	Fruit/spice	FFE033	5.2
*Bayeng*, *bedeneng*	Akwaya	*Lentinus squarrosulus* Mont.	Polyporaceae	Mushroom	FFE013	1.0
*Mango*	Mankon	*Mangifera indica* L.	Anacardiaceae	Fruits	FFE017	0.1
*Mangwah*, *manpang*	Bafut	*Mentha sp*.	Lamiaceae	Vegetable	FFE015	1.4
*Adondon*, *Suuh*	Bafut	*Monodora myristica* Dunal	Annonaceae	Spice	FFE022	2.5
*Tecjaw*	Widikum	*Moondia whitei* (Hook.f.) Skeels	Apocynaceae	Roots/fruit/spice	FFE043	0.7
*Funom*	Batibo	*Occimum basilicum* L.	Lamiaceae	Spice/vegetable	FFE046	2.7
*Fesong*, *efop*	Pining	*Occimum gratissimum* L.	Lamiaceae	Spice/vegetable	FFE031	2.5
*Begele*, *Bejabe*	Santa	*Passiflora edulis* Sims	Passifloraceae	Fruit	FFE059	0.4
*Azong grass*	Widikum	*Penisetum purpureum* Schumach	Poaceae	Vegetable	FFE056	0.2
Wild tomato	Bafut	*Physalis angulata* L.	Solanaceae	Fruits	FFE044	0.3
*Sop*, *Bush pepper*, *tone*	Bafanji	*Piper guineensis* Schumach. & Thonn.	Piperaceae	Spice	FFE029	1.6
*White pepper*	Bafut	*Piper nigrum* L.	Piperaceae	Spice	FFE045	2.5
*Feboh*	Mendakwe	*Pleurotus pulmonarius* (Fr.) Quel.	Pleurotaceae	Mushroom	FFE030	2.7
*Ankup*	Mankon	*Raphia farinifera* (Gaertn.) Hyl.	Arecaceae	Beverage, fruits	FFE047	4.2
*Djansang*, *lesah*	Ndop	*Ricinodendron heudelotii* (H.E.Baillon) J.B.Pierre ex E.M.Heckel.	Euphorbiaceae	Spice	FFE032	5.1
*Bush onion*	Bambili	*Scorodophleus zenkeri* Harms	Mimosaceae	Spice	FFE053	1.1
*Black jack*, *Njama njama*	Nkwen	*Solanum nigrum* L.	Solanaceae	Vegetable	FFE051	3.3
		*Solanum melongena* L.	Solanaceae	Spice	FFE023	0.9
*Sun flower*	Pidgin	*Helianthus annum* L.	Asteraceae	Vegetable	FFE040	0.1
		*Syzygium guineensis* (Wild.) DC.	Myrtaceae	Fruits	FFE035	0.5
*Nmborie*	Wum	*Talinum trianguare* (Jacq.) Wild.	Portulacaceae	Vegetable	FFE020	0.6
*Agreuh*	Ndop	*Termitomyces clypeatus* R. Heim	Tricholomataceae	Mushroom	FFE057	6.4
*Boh*	Widikum	*Termitomyces letestui* (Pat.) Heim.	Tricholomataceae	Mushroom	FFE024	4.1
*Efin*	Bambui	*Termitomyces* sp.	Tricholomataceae	Mushroom	FFE038	4.0
*Beteh*	Mankon	*Termitomyces ourantiacus* (R. Heim) R. Heim.	Lyophyllaceae	Mushroom	FFE036	2.2
*Eshuk*, *Ngon*	Nkwen	*Tetrapleura tetraptera* (Schumach. and Thonn)	Mimosaceae	Spice	FFE054	1.4
*Erita*	Bambili	*Vernonia amygdalina* Delile	Asperaceae	Vegetable	FFE049	2.2
*Atamah*	Santa	*Vernonia calvoana* (Hook.f.) Hook.f.	Asteraceae	Vegetable	FFE038	0.9
*Jensen*	Banso	*Vernonia guineensis* Benth.	Asteraceae	Roots/tubers	FFE058	0.5
*Ferebah*, *enkirgelinic*	Bambui	*Xylopia aethiopica* (Dunal) A. Rich.	Annonaceae	Spice	FFE052	3.8
*Etangebenut*	Santa	*Xylopia* sp.	Annonaceae	Spice	FFE048	0.3

### Local perception of the availability of wild edible plants and mushroom

**Fig. 3:**
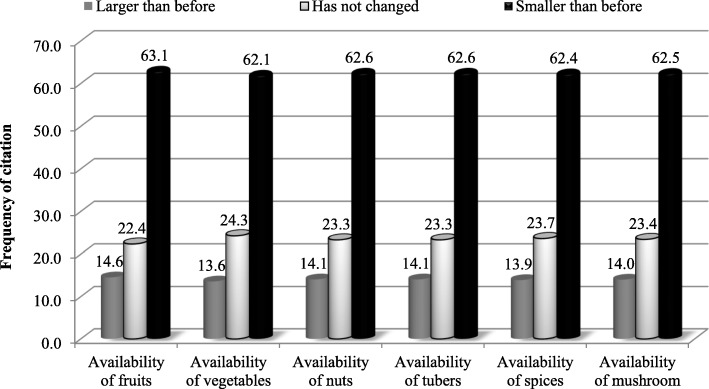
Respondent’s perception on availability of wild edible plants and mushrooms

Our results showed that wild edible plants in the Bamenda Highlands are under serious threat, as perceived by local informants. More than 60% of respondents believe availability of WEPM is getting smaller than before, while 23% think the availability has not changed, and 14% said availability is larger than before (Fig. 3).

Main reasons of scarcity of WEPM are excessive collection and other human activities including over-grazing, agricultural land expansion, uncontrolled bushfires, etc.

## Discussion

### Knowledge and use of wild edible plants and mushrooms in Bamenda Highlands

Cameroon is a culturally diverse country consisting of over 250 ethnic groups, and the cuisine significantly varies by ethnic group and region. Wild foods are essential components for these dishes and the regional cuisine. In the Bamenda Highlands of Cameroon, the Tikares appears to be the most populous ethnic group, and they were the first to settle on the Bamenda region [32]. According to anthropologists, they originated from northern Cameroon and migrated southwards and westwards in the eighteenth and nineteenth centuries to their current locations in the Western Grassfields (Bamenda Highlands) and Eastern Grassfields (Fumban) and the Tikar Plain of Bankim [33]. Despite the scarcity of ethnobotanical literature on wild foods, indigenous communities in this area have gathered wild edible plants and mushrooms for centuries as a strategy to complement their crop-livestock subsistence systems. Each community group has made particular choices among the wild food resources available, and this utilitarian relationship between indigenous communities and the WEPM can be contextualized in space and time. As it was described in temperate ecosystems, the dynamics of this relationship can vary depending on species availability, site accessibility, cultural acceptability, traditional ecological knowledge, migration, changes in lifestyle, and other socio-ecological processes [34]. These factors, according to [35], can determine the acceptability or a possible replacement of wild foods with modern foods.

In this study, 47 species were recorded. Some, including *Ricinodendron heudelotii*, *Piper guineensis*, *Cola* sp., *Tetrapleura tetraptera*, *Xylopia aethiopica*, and *Canarium schweinfurthii*, have been reported in previous surveys on non-timber forest products in Tikar Plain [36]. Others like bush mango (*Irvingia gabonensis*), njansang (*Ricinodendron heudelotii*), eru (*Gnetum africanum*), and kola nuts (*Cola* spp.) are also among the key non-timber forest products of Central Africa [37].

The number of species recorded during this survey does not certainly captures all the diversity of wild edible plants and mushrooms growing in this study area, as their availability rely on seasons. There is generally a relatively high importance of wild edible plants in the rainy season. The period of the survey coincided with the beginning of the rainy season when some species, although not yet providing edible parts, is re-sprouting, flowering, and fruiting. A cross-seasonal investigation will be required to capture the diversity of wild edible plants and mushrooms consumed in the study area.

Also, as argued by several authors, age, gender, and other sociocultural variables are likely to influence access to wild plant resources and the traditional ecological knowledge of wild foods [38].

### Nutritional potentials of wild edible plants and mushroom recorded

Several studies emphasize on the high nutritional importance of wild edible plants [39–42]. This is true for the species in different groups of WEPM recorded. Their seasonal relative importance greatly impacts the food and nutritional insecurity copying ability of households. Previous studies have confirmed that in times of food scarcity, they make human diets more diverse and add flavor, vitamins, and minerals [43].

#### Vegetables

The highly exploited wild vegetable *Gnetum* spp., locally called “eru” is very rich in proteins and minerals (Na, K, Ca, Mg, Fe) and contains all essential amino acids [44].

Waterleaf (*Talinum triangulare*) is reported to be very rich in carbohydrates, protein, steroid, oil, b-Carotene, crude fibers, and minerals like Ca, Mg, Na, and K [45].

Elemental analysis in mg/100 g (DW) of leaves of *Amaranthus hybridus* was done by previous studies [46]. These authors indicated that the leaves contained sodium (7.43), potassium (54.20), calcium (44.15), magnesium (231.22), iron (13.58), zinc (3.80), and phosphorus (34.91). The vitamin composition of the leaves in mg/100 g (DW) was as follows: carotene (3.29), thiamine (2.75), riboflavin (4.24), niacin (1.54), pyridoxine (2.33), ascorbic acids (25.40), and -tocopherol (0.50). These authors also reported 17 amino acids (isoleucine, leucine, lysine, methionine, cysteine, phenylalmine, tyrosine, threonine, valine, alanine, arginine, aspartic acid, glutamic acid, glycine, histidine, proline, and serine) detected in leaves of this species. Alkaloid, flavonoid, saponin, tannins, phenols, hydrocyanic acid, and phytic acid composition were 3.54, 0.83, 1.68, 0.49, 0.35, 16.99, and 1.32, respectively. These evidences are indication that the leaves of *Amaranthus hybridus* are important source of nutrients, minerals, vitamins, amino acids and phytochemicals, and low levels of toxicants.

For *Corchorus olitorius*, the proximate and mineral composition of leaves were investigated in Nigeria [47] and showed that the leaves contained 18.38 ± 0.32% ash, 12.54 ± 0.10% crude protein, 11.99 ± 0.50% crude lipid, and 19.56 ± 0.18% available carbohydrate. Their energy value reported was 200.78 ± 3.54 kcal/100 g. On the other hand, their mineral content comprises potassium (2814.15 ± 8.08 mg/100 g) and magnesium (76.69 ± 0.13 mg/100 g) as dominant elements, Na (54.56 ± 0.42 mg/100 g), Ca (30.55 ± 0.05 mg/100 g), P (6.68 ± 0.02 mg/100 g), Cu (2.52 ± 0.02 mg/100 g), Fe (19.53 ± 0.09 mg/100 g), Mn (5.95 ± 0.04 mg/100 g), and Zn (4.71 + 0.01 mg/100 g). These findings support the use of *Corchorus olitorius* leaves are rich sources of potassium, iron, copper, manganese, and zinc as well as high energy values essential in human nutrition. Antinociceptive/anti-inflammatory, anti-tumor, antipyretic, carminative, demulcent, laxative, stimulant, and stomachic properties were also reported for this species [48].

Strong free radical scavenging activity was reported for *Vernonia calvoana*, and the authors of this study concluded that *V*. *calvoana* could serve as source of strong dietary antioxidants [49]. Its amino acid composition compare favorably with that of WHO protein standard [50], and *Vernonia calvoana* is also rich source of carotenoids (between 30 and 41.5 mg/100 g DW), vitamin C (between 137.5 and 197.5 mg/100 g DW), and dietary fiber (24.9–30.1 g/100 g DW).

#### Spices

*Afrostyrax lepidophyllus* was investigated for its biological activity and phytochemical composition [51]. Using 3 different extracts, these authors reported tannin content of the order of 2.35 ± 0.3, 10.68 ± 0.1, 7, and 78 ± 0.2 mg eq Cat/g DM. That of anthocyanins were 0.79 ± 0.04, 0.65 ± 0.02, 1.65 ± 0.07, and 0.18 ± 0.03 mg eq C3GE/g MS. These findings support the antioxidant, anti-inflammatory, and anti-xanthine oxidase activity of *Afrostyrax lepidophyllus* seeds used in the human diet.

Fruits of *Mondia whitei* contain antioxidant vitamins C and E which had values of 14.50 mg/100 g and 2.45 μg/g, respectively [52]. Potassium and sodium are the most abundant mineral elements. The roots of this species are attracting the interests of cosmeceutical, nutraceutical, and pharmaceutical industries. They are traditionally used for the treatment of anorexia, stress, bilharzia, and sexual dysfunction as well as for general aches and pains. The efficacy of most of these claims have been analyzed several researchers who investigated in the biological activities *Mondia whitei* roots and reported antimicrobial, anti-inflammatory, and anthelmintic as well as aphrodisiac efficacy [53].

*Piper guineensis* is a rich source of calcium (179.52 ± 0.11 mg/100 g), potassium (98.52 ± 0.1011 mg/100 g), and phosphore (217.70 ± 0.41 mg/100 g), and vitamin B2 and C [54].

Chemical profiling of *Xylopia aethiopica* revealed the presence of different phytochemicals of various physiological and biological actions. The fruit was reported to contain 38.72 ± 0.61% fiber, 26.08 ± 1.41% carbohydrates, 18.47 ± 0.05% protein, 6.73 ± 0.01% lipid, 6.02 ± 0.84% moisture, and 4.00 ± 0.02% ash, and mineral analysis showed the abundance of some mineral elements in *Xylopia aethiopica* fruit like calcium, potassium, magnesium, sodium, irons, phosphorus, zinc, manganese, chromium, and copper [55]. It was also reported the presence of alkaloids, cardiac glycosides, saponins, tannins, flavonoids, polyphenols, and reducing sugars, vitamins A, C, and β-carotene, all bioactive substances that may be beneficial to health [56].

#### Fruits

Nutritional potential of *Canarium schweinfurthii* was investigated in Plateau State in Nigeria [57]. They indicated crude fat of the fruit as 64.04%, protein 6.39%, fibers 16.37%, and carbohydrates 3.85%, respectively. Mineral analysis revealed that phosphorus and sodium levels were 1.74 and 1.369 mg/100 g, respectively. These authors suggested that *Canarium schweinfurthii* is nutritive despite the presence of some low levels anti-nutritive components like oxalate. The final products will contain even less.

The presence of various phytochemical constituents like flavonoids, tannins, phenol, glycosides, fatty acids, and alkaloids was reported in the fruit of *Passiflora edulis* [58], as well as anti-inflammatory, anticonvulsant, antimicrobial, anticancer, antidiabetic, antihypertensive, anti-sedative, and antioxidant properties.

The leaves of *Physalis angulata* were investigated for their potentials in alleviating micronutrient deficiency [59]. The study found that *Physalis angulata* fruits have crude protein content of 10.97%, sodium 689.48 mg/100 g, and manganese 21.60 mg/100 g. Amino acid analysis indicated the presence of isoleucine, valine, phenylalanine, tyrosine, and leucine. The reported concentration of phytate/Zn supports its potential into food-based strategy to alleviate zinc malnutrition.

#### Roots and tubers

The antiprostate cancer and antiangiogenic activity of the roots of *Vernonia guineensis* were demonstrated, supporting the use of the tubers of this plant for the treatment of prostate cancer [60].

#### Beverage

Records from the database of Plant Resource of Tropical Africa (PROTA) indicate that the fruit pulp of *Raphia farinifera* contains about 24% oil. The major fatty acids in seed oil are palmitic acid, oleic acid, and linoleic acid. The main sterol is β-sitosterol. The fruit pulp has shown antibacterial activity against *Staphylococcus aureus* but not against the gram-negative bacteria *Escherichia coli*, *Pseudomonas aeruginosa*, and *Salmonella typhi*; it also had no activity against the fungi *Candida albicans* and *Aspergillus niger*.

It is thus clear from various screening of the recorded plants that almost all of them are important from nutraceutical points. Many of the recorded plants are of rich nutritional value as sources of micro and macro elements, roughage, protein, and amino acids without anti-nutritional factors [61]. Local communities in the Bamenda Highlands thus derive important nutrients from these plants. However, some are lacking scientific nutritional knowledge, and many of their values remain either uninvestigated or undocumented because their products are used locally without being reflected in national or international markets. Therefore, systematic documentation of indigenous knowledge regarding the identity and use of wild edible plants is an urgent concern because both biological resources and indigenous knowledge are diminishing with high destruction and a growing disinterest among the younger generation.

## Conclusion

With respect to our stated hypothesis, we recorded 47 species of wild edible plants and mushrooms commonly gathered by the local population in the Bamenda Highlands use. Overall, the study shows that these species are largely consumed by local populations in the study area and play important role in household diets. They have great potential to contribute to food and nutritional security. However, for some of the recorded species, many of their values remain either uninvestigated or undocumented. In addition, the status of many of the commonly gathered and eaten species is declining in study area, driven by deforestation, bushfires, and over harvesting. This study strongly recommends actions aimed at promoting the conservation of wild edible plants as part of food security strategy by households. Sound nutrients and metabolites profiling of poorly known species need to be investigated to enhance their contribution in addressing food insecurity. Further, it is recommended to continue investigating on the factors that may determine the knowledge and use of wild edible plants.

## Data Availability

Plant specimens were deposited in the Département de Biologie et Physiologie Végétale Université de Douala.
